# YAP1 overexpression is associated with poor prognosis of breast cancer patients and induces breast cancer cell growth by inhibiting PTEN

**DOI:** 10.1002/2211-5463.12597

**Published:** 2019-02-14

**Authors:** Liwen Guo, Yutang Chen, Jun Luo, Jiaping Zheng, Guoliang Shao

**Affiliations:** ^1^ Department of Interventional Radiology Zhejiang Cancer Hospital Hangzhou China

**Keywords:** AKT, apoptosis, breast cancer, Hippo, PTEN, YAP1

## Abstract

YES‐associated protein 1 (YAP1) plays a key role as a transcriptional coactivator in the Hippo tumor suppressor pathway. YAP1 is overexpressed in a variety of cancers and is considered to be encoded by a proto‐oncogene. However, the role of YAP1 remains debatable, because both gain and loss of YAP1 expression have both been reported in breast cancer (BC). Here, we found that elevated expression of YAP1 mRNA in BC was negatively correlated with relapse‐free, distant metastases‐free and overall survival rates. We then knocked down or overexpressed YAP1 in human BC cells, and examined cell proliferation, apoptosis, and tumorigenic ability *in vivo*. We identified that YAP1 promotes cell growth and inhibits cell apoptosis of BC through the phosphatase and tensin homolog deleted on chromosome 10–AKT signaling pathway, and thus suggest that YAP1 might serve as a new target for inhibiting BC progression.

**Abbreviations**
BC: breast cancer
CCK8: cell counting kit‐8
GAPDH: glyceraldehyde‐3‐phosphate dehydrogenase
PTEN: phosphatase and tensin homolog deleted on chromosome 10
RNAi: RNA interference
siRNA: small interfering RNA
YAP1: YES‐associated protein 1

Breast cancer (BC) is among the most prevalent malignancies and is now the second‐leading cause of cancer death in privileged societies (198 000 deaths; 15.4% of total cancer deaths in these countries) and is therefore a major health issue for women 1, 2, 3. Despite numerous advances in prevention, surgical resection, radiotherapy, and chemotherapy, the incidence of BC continues to rise. Due to the high incidence of postoperative recurrence and metastasis, the prognosis of BC patients is still dismal. Millions of women worldwide die of BC each year 4, 5. Thus, a better understanding of the molecular basis of the genesis of BC is urgently needed to design targeted, molecularly based therapies.

The Hippo signaling pathway was discovered in *Drosophila* flies in the 1990s by the *Drosophila* genetic screen, which is used to display mutations that show excessive tissue growth. This signaling pathway plays a key role in organ growth control, regeneration, and tumor inhibition 6, 7. YES‐associated protein 1 (YAP1) is a pivotal effector of the Hippo signaling pathway. YAP1 enhances gene transcription activity by binding to transcription factors, such as transcription enhancers and runt‐domain transcription factors (Runx) 8, 9. Among these factors, most belong to apoptosis suppressor or growth promoter genes. It is reported that the upregulation of YAP1 is often observed in many human cancers, which suggests that it may be a potent drug target and worthy of further study 10, 11, 12.

In the current study, we assessed the prognostic relevance of YAP1 mRNA expression in BC patients through meta‐analysis of gene expression profiles from 4142 patients with BC using a Kaplan–Meier plotter (an online tool), studied the effect of YAP1 on cell proliferation and apoptosis in BC cell lines, and explored its potential mechanism.

## Materials and methods

### Kaplan–Meier plotter online survival analysis

A Kaplan‐Meir plotter was used that was able to assess the effect of 22 277 genes on survival in 4142 BC patients 13. A background database was established using gene expression data and survival information downloaded from Gene Expression Omnibus (Affymetrix microarrays only), the Cancer Genome Atlas, and the European Genome‐phenome Archive. The database is handled by a PostgreSQL server, which integrates gene expression and clinical data simultaneously. Briefly, YAP1 (213342_at) is entered into the database; relapse‐free survival, distant metastasis‐free survival, or overall survival is selected as the survival endpoint; the Auto select best cutoff option is checked (for earlier releases of the database, 2014 is selected from the drop‐menu). Kaplan–Meier survival plots are then obtained. The number‐at‐risk, hazard ratios (and 95% confidence intervals) and log‐rank *P* values were calculated and displayed on the main plot.

### Cell culture and antibodies

Human BC cell lines were purchased from the American Type Culture Collection (Manassas, VA, USA). Cells were cultured in RPMI‐1640 medium supplemented with 10% heat‐inactivated fetal bovine serum (Hyclone, South Logan, UT, USA) in a humidified incubator containing 5% CO_2_ at 37 °C. Cells in the exponential growth phase were used for all experiments. The antibodies used in this study, including anti‐YAP1 (cat. no. 4912; 1 : 1000 dilution), anti‐phosphatase and tensin homolog deleted on chromosome 10 (p‐PTEN) (cat. no. 9559; 1 : 1000 dilution), anti‐p‐PTEN (cat. no. 9554; 1 : 1000 dilution), anti‐AKT (cat. no. 9272; 1 : 1000 dilution), anti‐p‐AKT (cat. no. 13038; 1 : 1000 dilution), and anti‐glyceraldehyde‐3‐phosphate dehydrogenase (GAPDH) (cat. no. 5174; 1 : 1000 dilution), were purchased from Cell Signaling Technology Inc. (Beverly, MA, USA).

### Cell lysate preparation and western blot analysis

Cells were scraped and lysed using lysis buffer (50 mm Tris/HCl pH 7.4, 0.5% sodium deoxycholate, 1% Nonidet P‐40, 150 mm NaCl, 0.1% sodium dodecyl sulfate, and 0.02% sodium azide) on ice for 15 min and then debris was removed by centrifugation (16 128 ***g***, 15 min). Cell extracts were boiled in loading buffer for 5 min, and then an equal volume of cell extract was separated on 10% SDS/PAGE gels, then transferred to polyvinylidene fluoride membranes (Millipore, Billerica, MA, USA). The membranes were blocked for 1 h in 5% non‐fat milk in Tris‐buffered saline solution and Tween buffer (10 mmol·L^−1^ Tris/HCl, 0.05% (w/v) Tween 20, and 0.5 mol·L^−1^ NaCl), and then membranes were incubated at 4 °C overnight with primary antibodies. The relative protein levels in different samples were normalized to the GAPDH concentration. The resulting immunoreactive bands were visualized using an enhanced chemiluminescence substrate system (Millipore). Each experiment was repeated at least three times.

### PC3.1/YAP1 plasmid and transfection

A pcDNA3.1/YAP1 plasmid and the vector were designed and synthesized by GenePharma (Shanghai, China). The plasmid was transfected into cells using the Lipofectamine 3000 (Invitrogen, Carlsbad, CA, USA) following the manufacturer's protocols. In brief, 0.5 μg plasmid was mixed with 25 μL Opti‐MEM, 1 μL Lipofectamine 3000 was mixed with 24 μL Opti‐MEM, and the transfection mix was made by mixing the plasmid preparation solution with an equal volume of the Lipofectamine 3000 solution. After being placed at room temperature for 20 min, the transfection mix solution was added into the cell cultured plates and mixed gently. Subsequently, the cells were incubated for 4 h at room temperature and 5% CO_2_. The transfection medium was then replaced by complete medium.

### YAP1 siRNA transfection

Small interfering RNA (siRNA) against YAP1 was designed and synthesized by GenePharma. The cells were seeded in six‐well plates, allowed to grow overnight to reach 70–80% confluence, and transfected with the siRNA recombinants (30 nm) using Lipofectamine RNAiMAX (Life Technologies, Carlsbad, CA, USA) regent. The siRNA sequences chosen to target YAP1 were RNAi‐1: 5′‐GGUGAUACUAUCAACCAAATT‐3′, RNAi‐2 5′‐GACGACCAAUAGCUCAGAUTT‐3′, in non‐conserved regions of the YAP1 open reading frame (GenBank accession number NM_006106). blast (GenePharma, Shanghai, China) analysis shows no homology of the RNAi sequences to any other sequence in the Human Genome Database. Scrambled RNAi, 5′‐UUCUCCGAACGUGUCACGUTT‐3′, was also obtained from GenePharma as a negative control. The YAP1 gene knockout efficiency was confirmed by western blot analysis.

### Apoptosis detection assay

An annexin V‐fluorescein isothiocyanate (FITC) kit (KeyGen Biotech. Co. Ltd., Nanjing, China) was used to detect apoptosis. Three days after transfection, the cells were harvested, then washed twice using ice‐cold PBS. According to the manufacturer's instructions, the cells were stained with binding buffer containing annexin V–FITC and propidium iodide in the dark at room temperature for 15 min. Finally, the apoptosis ratio was analyzed by flow cytometry within 1 h.

### Cell proliferation assay

A cell counting kit‐8 (CCK‐8) cell viability assay kit (Dojindo Laboratories, Kumamoto, Japan) was used to determine the cell proliferation. In brief, cells were incubated in a 96‐well plate with 5 × 10^3^ cells/well for 48 h. Then, we added 10 μL of cell viability assay kit solution to each well. After 2 h incubation at room temperature without light, the absorbance of each well was measured at 450 nm using a microplate reader (ELx800; Biotek, Winooski, VT, USA).

### Xenograft tumor model

BALB/c nude mice (4–5 weeks old, 18–20 g) were purchased from Zhejiang University Laboratory Animal center (Hangzhou, Zhejiang, China). The mice were divided into four groups of five mice each. Each mouse was injected on the mammary pads with RNAi‐1‐ or RNAi‐2‐treated cells (5 × 10^6^) on the right side and with the same number of vector‐transfected cells on the left side. On day 42, the mice were killed, and the tumors were excised and weighed. All animal experiments conformed to the *Guide for the Care and Use of Laboratory Animals* (National Research Council, National Academies Press, Washington, DC, USA, 2011), and were conducted following protocols approved by the Ethics Committee of Zhejiang cancer Hospital.

### Statistical analysis

The results are represented by the mean ± SD. Student's *t* test or one‐way ANOVA was used to determine the significance of differences between various experimental groups (prism v.5.0, GraphPad Software Inc., La Jolla, CA, USA). *P* level of < 0.05 (*P <* *0.05; **P <* *0.01) was considered to be statistically significant.

## Results

### YAP1 is upregulated in BC cells and correlated with poor prognosis of BC patients

**Figure 1:**
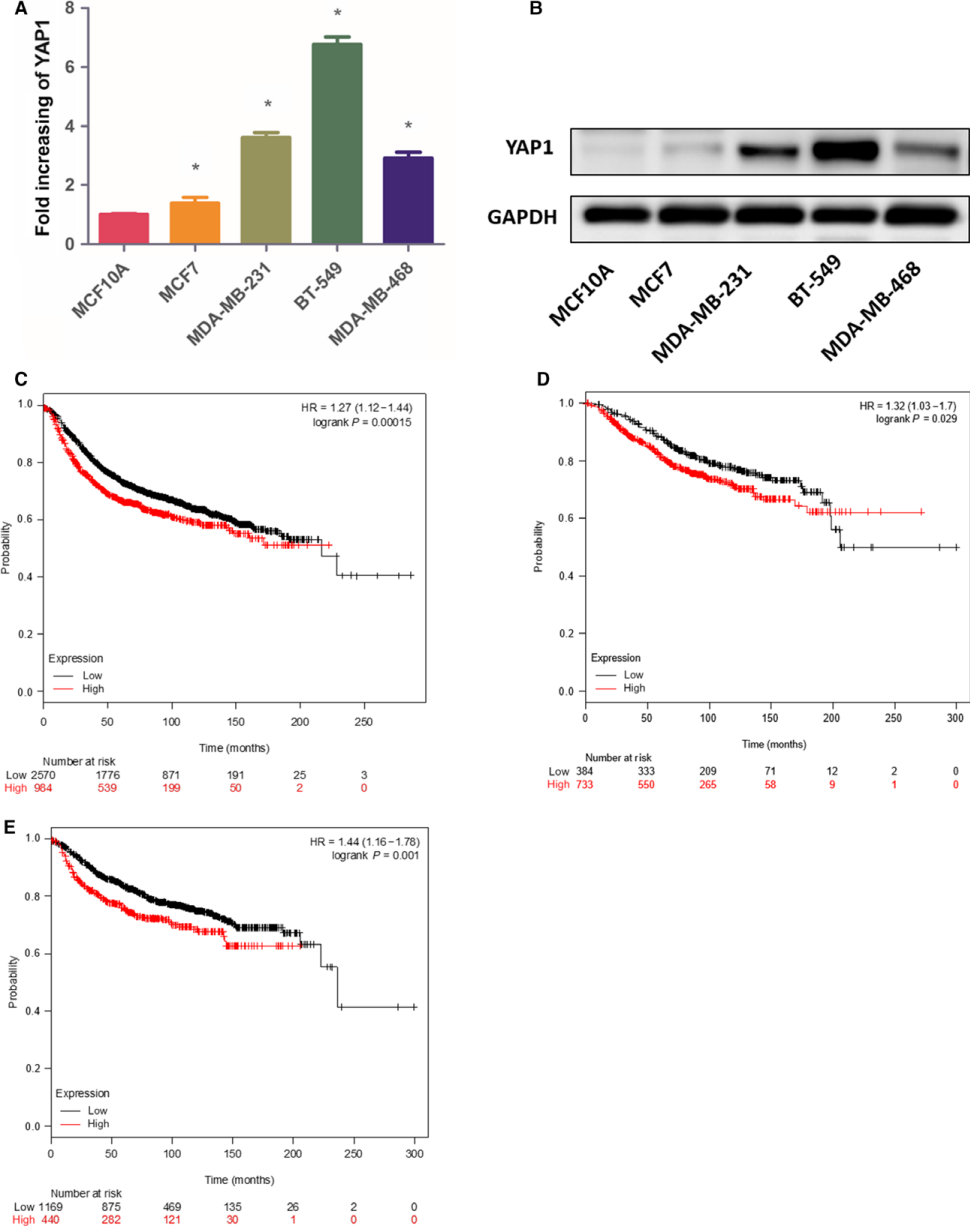
YAP1 is upregulated in BC cells and correlated with poor prognosis of BC patients. (A,B) Western blotting of YAP1 expression in normal breast epithelial cells and BC cells. (C–E) Kaplan–Meier plots showing the association between YAP1 mRNA expression and patients’ relapse‐free survival (C), distant metastases‐free survival (D), and overall survival (E). Data are presented as mean ± SD of three biological replicates and are analyzed by two‐tailed Student's *t* test; *P <* *0.05 *vs* MCF10A. HR, hazard ratio.

The results of western blotting showed YAP1 was dramatically upregulated in cultured BC cells (MCF7, MDA‐MB‐231, BT‐549, MDA‐MB‐468) compared with that in normal breast epithelial cells (MCF10A) (*P *<* *0.05; Fig. 1A,B).

In an online survival analysis with the Kaplan–Meier plotter, the prognostic value of YAP1 (Affy id: 213342_at) mRNA was demonstrated. It was shown that elevated YAP1 mRNA expression had negative effects on the patients’ relapse‐free survival (hazard ratio, 1.27; *P *=* *1.5 × 10^−4^), distant metastases‐free survival (hazard ratio, 1.44; *P *=* *0.001), and overall survival (hazard ratio, 1.32; *P *=* *0.029) rates. Increased expression of YAP1 mRNA was found to carry an adverse prognostic value (Fig. 1C–E).

### YAP1 modulates proliferation of BC cells

**Figure 2:**
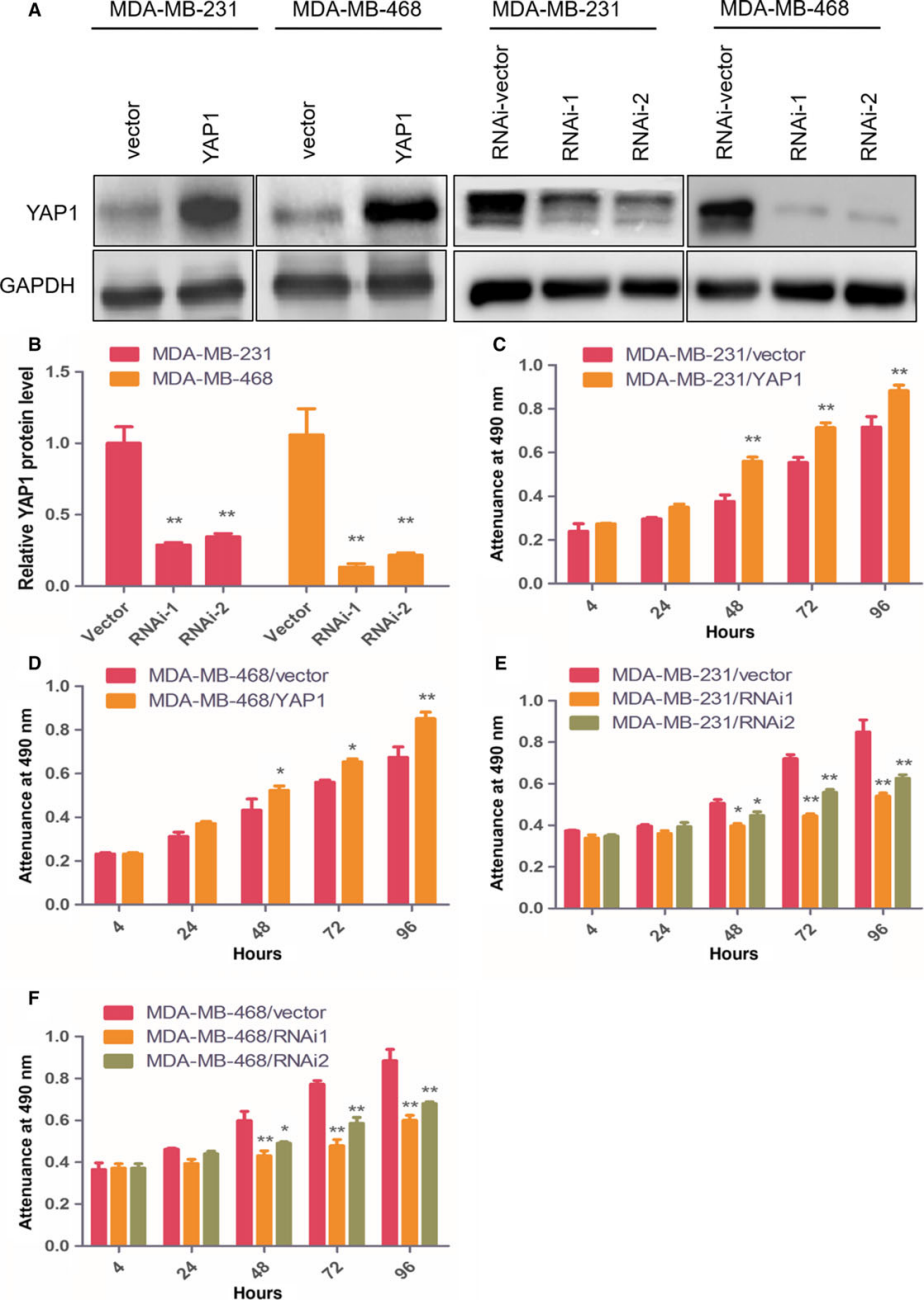
YAP1 modulates proliferation of BC cells. (A) Western blot of indicated BC cells transfected with YAP vector, YAP1, YAP1‐RNAi vector, YAP1‐RNAi1 or YAP1‐RNAi2. (B) Protein quantification of the western blot results. (C,D) CCK8 assays revealed that overexpression of YAP1 significantly increased the growth rate of indicated cells. (E,F) CCK8 assays revealed that downregulation of endogenous YAP1 significantly reduced the growth rate. Data are presented as mean ± SD of three biological replicates and are analyzed by two‐tailed Student's *t* test; *P <* *0.05 *vs* vector, **P <* *0.01 *vs* vector.

To understand the effect of YAP1 on the proliferation of BC cells, we used siRNA to reduce YAP1 expression in the MDA‐MB‐231 and MDA‐MB‐468 BC cells. The siRNAs were designed to target the YAP1 messenger RNA (mRNA). blast analysis shows no homology of the siRNA sequences to any other sequence in the Human Genome Database, including other members of the YAP1 gene family. We also overexpressed YAP1 in MDA‐MB‐468 and MDA‐MB‐231 cells. The expression level of YAP1 was verified by western blot (Fig. 2A,B). CCK‐8 assays revealed that overexpression of YAP1 significantly increased the MDA‐MB‐231 and MDA‐MB‐468 cell numbers at 48 h after plating compared to vector control cells (Fig. 2C,D), while knockdown of YAP1 inhibited the proliferation of BC cells significantly (Fig. 2E,F).

### YAP1 regulates the tumorigenesis of BC

**Figure 3:**
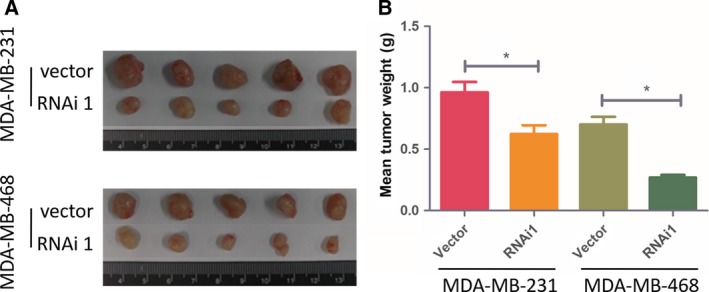
(A) Images of excised tumors from five BALB/C nude mice at 42 days after injection with YAP1 RNAi1‐transfected cells and vector transfected cells. (B) Average weight of excised tumors. Data are presented as mean ± SD and are analyzed by two‐tailed Student's *t* test (*n* = 5 per group); *P <* *0.05 *vs* vector.

To validate the effects of YAP1 on cell proliferation assays *in vitro*, we established a BC xenograft model in nude mice with MDA‐MB‐231 and MDA‐MB‐468 cells. As revealed in Fig. 3A, knockdown of YAP1 significantly reduced the ability of tumorigenicity in the nude mice. The final xenograft tumor weights in the YAP1‐silenced groups were significantly lower than that in the control groups (Fig. 3B). Taken together, these findings indicate that YAP1 play an important role in BC cell proliferation *in vitro* and tumorigenicity *in vivo*.

### YAP1‐induced PTEN loss leads to increased AKT signaling

**Figure 4:**
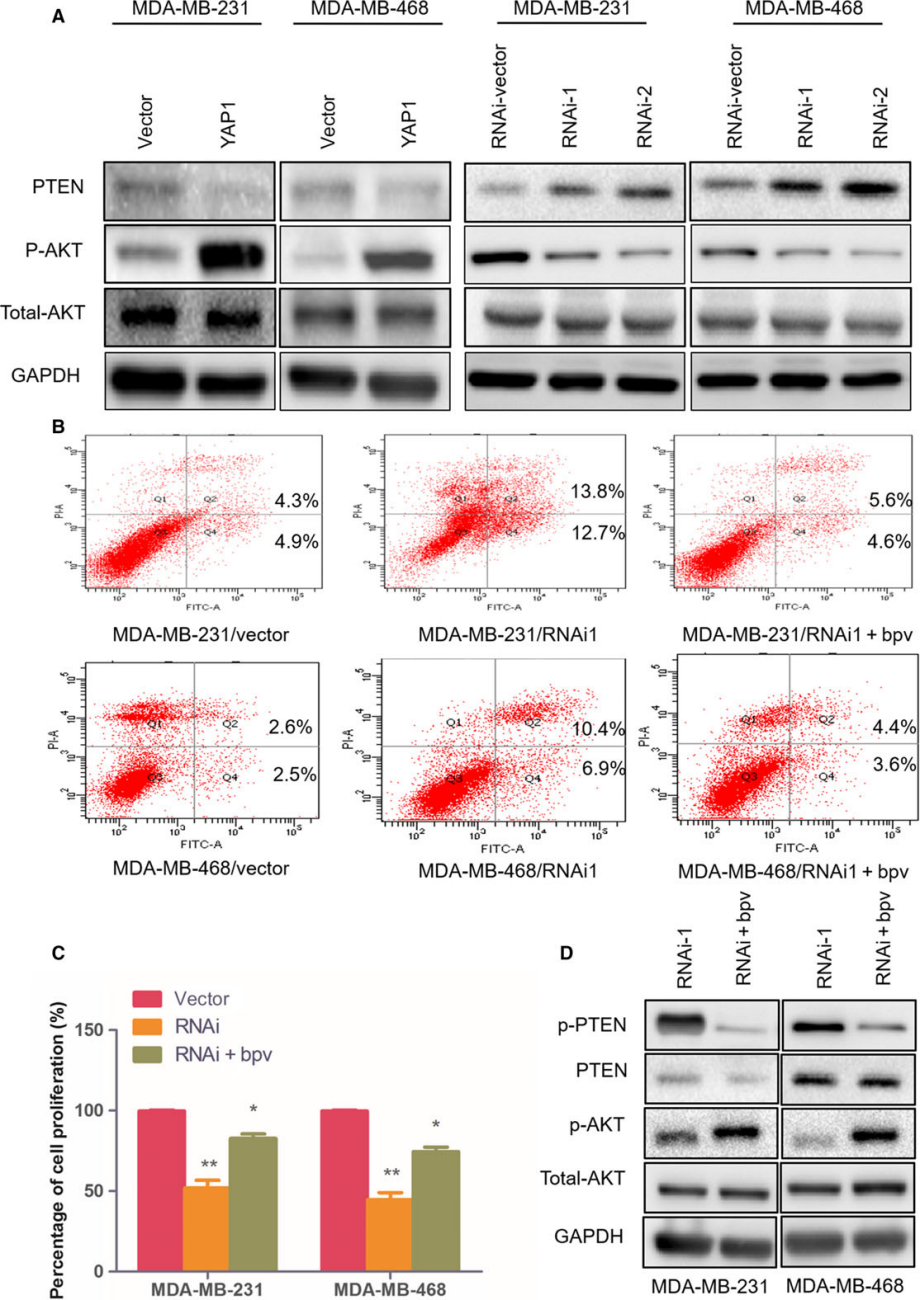
YAP1 affects cell apoptosis and proliferation through regulation of PTEN–AKT signaling. (A) Western blot analysis of PTEN, phosphorylated AKT (p‐AKT), and total AKT protein in the indicated BC cell lines. (B) Flow cytometric assays revealed the role of PTEN‐specific inhibitor bpV(HOpic) in the apoptosis of YAP1‐RNAi1‐transduced cells. (C) CCK8 assays revealed the role of bpV(HOpic) in the proliferation of YAP1‐RNAi1‐transduced cells. (D) Western blot analysis of p‐PTEN, PTEN, p‐AKT, and total AKT protein in bpV (HOpic)‐treated YAP1 silenced cells. Data are presented as mean ± SD of three biological replicates and were analyzed by two‐tailed Student's *t* test; *P <* *0.05 *vs* vector, **P <* *0.01 *vs* vector.

The PTEN–AKT signal pathway plays an important role in cell proliferation. Therefore, we explored whether YAP1 induction regulated PTEN–AKT activation in BC cells. As shown in Fig. 4A, PTEN was decreased in YAP1‐overexpressing cells but increased in YAP1‐silenced cells. Consistently, the phosphorylation of AKT (p‐AKT), a common downstream target of PTEN, was increased in YAP1‐overexpressing cells and decreased in YAP1‐silenced cells, suggesting that the change in PTEN–AKT signaling pathway activity is modulated by YAP1.

To further uncover the important role of PTEN in the tumorigenesis and proliferation regulated by YAP1, the PTEN‐specific inhibitor bpV(HOpic) (Selleckchem, Houston, TX, USA) was added in the cell culture medium for 72 h (2 μm). As shown in Fig. 4B and Table 1, results of flow cytometry assays revealed that the inhibition of PTEN decreased the percentage apoptosis and promoted cell proliferation (Fig. 4C). Results of the western blot assays also showed bpV(HOpic) inhibited the phosphorylation of PTEN; accordingly, the levels of p‐AKT protein rapidly increased (Fig. 4D). In conclusion, these findings revealed that the PTEN–AKT pathway is associated with the regulation of cell proliferation caused by overexpressing or silencing YAP1 in BC cells.

**Table 1:** The effects of YAP1 knockdown and bpV on apoptosis of BC cells (Q2 + Q4)% (*n* = 3). Data are presented as mean ± SD of three biological replicates. The difference in each group was tested for significance using one‐way ANOVA

	Vector	RNAi1	RNAi1 + bpV	*F*	*P*
MDA‐MB‐231	9.43 ± 0.45	26.13 ± 0.81^a^	10.5 ± 0.51^a^^,^ b	237.2	<0.001
MDA‐MB‐468	5.80 ± 0.41	17.37 ± 0.52^a^	8.13 ± 0.38^a^^,^ b	195.0	<0.001

^a^ *P* < 0.01 compared with the vector control in the same row. ^b^ *P *< 0.01 compared with the RNAi group in the same row.

## Discussion

The Hippo signaling pathway regulates cell number by modulating cell proliferation, apoptosis, and differentiation in both *Drosophila* and mammals 14. The core part of the *Drosophila* Hippo signaling pathway is a protein kinase cascade formed by four proteins: Wart, Salvador, Hippo, and Mats, in which Hippo, facilitated by Salvador, phosphorylates and activates Wart and Mats. In *Drosophila*, Yorkie is the main substrate of Wart. Yorkie inactivated by mutation or phosphorylation leads to tissue atrophy, whereas upregulation of Yorkie leads to excessive tissue hyperplasia 15.

YAP1 is the homologue of Yorkie in the mammalian system. It was first identified due to its binding to the SH3 domain of Src protein tyrosine kinases and Yes proto‐oncogene product 16. In humans, the *YAP1* gene encodes a 65 kDa proline‐rich phosphoprotein. YAP1 mRNA is expressed in a wide range of tissues and throughout the whole process of development 17. Numerous studies have shown that overexpression of the *YAP1* gene is found in various human cancers. YAP1 can induce epithelial–mesenchymal transition, increase the number of cancer stem cells and inhibit cell apoptosis *in vitro*, and the abilities of cancer cell invasion, migration, and tumorigenicity in nude mice can be reduced by *YAP1* knockdown 15, 18, 19, 20.

However, some researchers have suggested that YAP1 might function as a tumor suppressor. Yuan *et al*. 21 reported that knockdown of YAP1 in breast cell lines suppressed anoikis, increased migration, and invasiveness and enhanced tumor growth in nude mice. Barry *et al*. 22 demonstrated that YAP1 is significant silenced in a highly aggressive and undifferentiated human colorectal carcinoma, and that its expression can restrict the growth of colorectal carcinoma xenografts. YAP1 is also an important component of c‐Jun‐mediated apoptosis 23.

Our findings indicate greater expression of YAP1 mRNA in BC tissues than in normal breast tissues and a negative correlation with patient survival. Consistently, we found YAP expression was increased in BC cell lines more than that in normal breast epithelial cells. We chose MDA‐MB‐231 cell line and MDA‐MB‐468 cell line as the study cells for subsequent overexpression or knockout YAP1 tests because the expression of YAP1 was significantly increased in these cell lines. The results of the proliferation test showed that overexpression of YAP1 promotes cell growth of BC cells *in vitro*, while YAP1 knockdown significantly inhibits the cell growth as well as tumorigenicity *in vivo*. These results indicate that YAP1 promotes growth and proliferation of BC. By western blot assays, we found that the PTEN protein level was significantly reduced after YAP1 was overexpressed. PTEN, encoded by a well‐known tumor suppressor gene, is a negative regulator of the phosphoinositide 3‐kinase–AKT pathway; this pathway is a pivotal carcinogenic pathway that regulates cell proliferation and apoptosis. To determine the role of PTEN in AKT activation induced by YAP1 expression, we changed the expression of YAP1 in MDA‐MB‐231 and MDA‐MB‐468 cells; we found that the loss of YAP1 increased PTEN and decreased AKT phosphorylation. Consistently, overexpressed of YAP1 induced significantly higher phosphorylation of AKT. bpV(HOpic), a specific inhibitor of PTEN, can rescue apoptosis, as well as growth inhibition, induced by YAP1 knockdown in BC cells.

In conclusion, our data demonstrate that elevated YAP1 mRNA expression had a negative effect on the survival rate of patients with BC. Knockdown of YAP1 suppressed proliferation and induced apoptosis of BC cells. In research into the mechanism, we found that PTEN activity and the levels of active AKT were affected by the expression level of YAP1. YAP1 may be a novel target for effective inhibition of BC progression, and this study may shed light on the possible benefits of YAP1 inhibition in BC clinical treatment.

## Conflict of interest

The authors declare no conflict of interest.

## Author contributions

LG and YC performed experiments and drafted the manuscript. JL carried out the cell proliferation assay and apoptosis assay. JZ performed the statistical analysis and was involved in data discussion. GS designed and supervised the experiments, and proofed the manuscript. All authors have read and approved the final draft of the manuscript.
